# Clinical Features of Extragastrointestinal Stromal Tumor Compared with Gastrointestinal Stromal Tumor: A Retrospective, Multicenter, Real-World Study

**DOI:** 10.1155/2021/1460131

**Published:** 2021-12-13

**Authors:** Huolun Feng, Weixian Hu, Chengbin Zheng, Wei Wang, Guoliang Zheng, Xingyu Feng, Wenjun Xiong, Guosheng Lin, Yongjian Zhou, Yan Zhao, Yong Li

**Affiliations:** ^1^Department of General Surgery, Guangdong Provincial People's Hospital, Guangdong Academy of Medical Sciences, Guangzhou, Guangdong 510080, China; ^2^The Second School of Clinical Medicine, Southern Medical University, Guangzhou, Guangdong 510515, China; ^3^Department of General Surgery, Jieyang People's Hospital, Jieyang Affiliated Hospital, Sun Yat-Sen University, Jieyang 522000, China; ^4^Department of Gastrointestinal Surgery, Guangdong Provincial Hospital of Chinese Medicine, The Second Affiliated Hospital of Guangzhou University of Chinese Medicine, Guangzhou 510120, China; ^5^Department of Gastric Cancer, Cancer Hospital of China Medical University, Liaoning Cancer Hospital and Institute, Shenyang, Liaoning 110042, China; ^6^Department of Gastric Surgery, Fujian Medical University Union Hospital, Fuzhou, Fujian 350001, China

## Abstract

**Objective:**

This study aimed to compare the tumor characteristics and long-term outcomes between EGIST and GIST. The confounding function was applied to improve the result credibility in the case of small sample size. *Design, Setting, and Participants*. This cohort study enrolled 55 patients with EGIST who underwent surgery and were selected from four high-volume hospitals in China and 221 GIST patients who were collected from one of the four hospitals between January 2006 and September 2017. We used propensity score matching (PSM) and subgroup analysis to compare EGIST with GIST in terms of prognosis. The confounding function was used for sensitivity analysis to reduce unmeasured confounding.

**Results:**

We matched 43 patients in each of the GIST and EGIST groups by PSM. We compared EGIST data with GIST data to explore the prognostic factors between them. In the multivariate Cox regression model, tumor location of EGIST was negatively correlated with overall survival (after PSM: HR, 4.32; 95% CI, 1.22–15.26) or disease-free survival (after PSM: HR, 9.79; 95% CI, 2.22–43.31), which was also intuitively shown in the Kaplan–Meier survival curves (all *P* values < 0.05). In the subgroup analysis, EGIST with high risk factors had a worse prognosis than GIST. In unmeasured confounding analysis, the overall curve tends to show all combinations of c(0) of c(1) up to 2.0, none of which would bring the corrected relative risk to 1 for OS and DFS. *Conclusions and Relevance*. EGIST was associated with worse prognosis compared with GIST patients, particularly in EGIST patients with high risk factors, while there was a similar prognosis without those high risk factors.

## 1. Introduction

Extragastrointestinal stromal tumor (EGIST) is mainly located in the nongastrointestinal tract and is rarer compared to the gastrointestinal stromal tumor (GIST) [1]. EGIST is identified by meeting the standard diagnosis of GIST pathology, immunohistochemistry, and molecular gene analysis. Some studies have suggested that EGIST may be a distant or implantation metastasis of GIST [2, 3], while others have shown that EGIST is a particular subtype of GIST [4, 5]. Those studies [2–6] also focused on exploring whether EGIST was a secondary tumor. Regarding the clinicopathologic characteristics, several previous studies have sought the difference of EGIST from GIST using only the GIST data [7–13], while others have illustrated EGIST features without those data [2–6, 14]. However, those have suggested that EGIST was negatively associated with prognosis. The standard treatment for GIST is radical surgical resection, which is combined with adjuvant imatinib therapy for cases classified as medium or high risk based on the NIH criteria [15]. However, the therapeutic strategy of EGIST is with reference to the treatment of GIST and unfortunately has poor prognosis in previous studies [2–6].

Many factors play an important role in disease-free survival (DFS) and overall survival (OS) from GIST, which are routinely used to stratify tumor risk and guide treatment [1, 7–13]. These factors often include tumor size, tumor necrosis, tumor mitosis, and adjuvant therapy [16, 17]. Whether those factors can be applied to the EGIST or affect the EGIST prognosis is debatable [2–6, 14], possibly due to the low incidence and short OS in EGIST. However, previous studies failed to compare EGIST with GIST to assess the role of these indicators. EGIST is currently an incompletely researched tumor; thus, many treatments were based on the GIST model. The lack of exploration of the clinicopathological differences between EGIST and GIST may lead to improper treatment, or it may have caused the poor prognosis of EGIST.

There is an urgent need to explore the clinicopathological differences between EGIST and GIST and to conduct subgroup analysis according to different factors. Thus, this study aimed to provide some evidence for the treatment of EGIST by referring to the GIST measure, which may effectively evaluate the disease and choose different therapeutic methods.

## 2. Methods

The Institutional Review Board of the Guangdong Province People's Hospital approved this study and deemed that separate informed consent was not necessary. There were 62 EGIST cases from four centers and 570 GIST cases from the Guangdong Province People's Hospital obtained between January 1, 2006, and September 31, 2017. EGIST was retrospectively identified from four major centers in China, which included the Guangdong Provincial People's Hospital, Liaoning Cancer Hospital & Institute, Guangdong Province Traditional Medical Hospital, and Fujian Medical University Union Hospital. A total of 570 patients who underwent complete resection and had complete clinicopathological and follow-up data of primary GIST were collected in the present study, while those with recurrent or metastatic disease were excluded.

The inclusion and exclusion criteria and molecular characteristics of EGIST were consistent with those of our previous study [14]. In our previous study, “the criteria for inclusion were as follows: (1) having pathologically confirmed diagnosis of EGIST, (2) not having radiological and perioperative evidence of other primary lesions, (3) undergoing surgical treatment, and (4) having no record of surgical laparotomy of the entire abdomen for other lesions. And then the exclusion criteria were as follows: (1) microscopically identified adhesion between the tumor and the gastrointestinal serosa, (2) prior history of GIST/EGIST, (3) the presence of other malignancies, and (4) death caused by other diseases [14].

We included demographic and clinicopathological data such as age, sex, tumor size, tumor location (including EGIST and GIST), surgical time, surgical bleeding, Eastern Cooperative Oncology Group (ECOG) score, tumor necrosis, tumor mitosis, histopathological classification, and adjuvant therapy. Because of the limited number of EGIST cases, some factors were translated into categorical variables according to previous applications. Some factors such as adjuvant therapy, tumor size, tumor necrosis, and tumor mitosis had an important prognostic effect in GIST, which was defined as a high risk factor. Meanwhile, tumor recurrence was diagnosed based on clinical, radiological, or second surgery. We showed the immunohistochemical and molecular gene data in Supplementary File 1 because it was used to identify the basis for the diagnosis of EGIST or GIST, but the current study mainly focused on clinical prognosis comparison. OS was calculated from the date of surgery to the last follow-up date (May 31, 2018) or death, while DFS was determined from the date of surgery to disease recurrence or death. Disease recurrence was determined based on radiological (CT/MRI) evidence. Outpatient surveillance or telephone calls were used to follow up. The outpatient postoperative follow-up included clinical, laboratory examinations and computed tomography scanning, performed every 3 months for the first 2 years, every 6 months from the 3^rd^ to 5^th^ years, every 12 months thereafter or earlier as deemed necessary by the patient treating physician based on their conditions [14].

EGIST was diagnosed by two processes. Firstly, a surgeon with less than 10-year experience in abdominal surgery assessed and resected EGIST, if there are no distant metastases and isolated tumor lesions. A surgeon did carefully check the patients in resection of the tumor, and all suspicious lesions were sent for pathological examination at least to ensure that the tumor is primary under the condition of the naked eye and existing diagnostic examination. Secondly, the pathologist handled the specimen through formalin fixed in paraffin-embedded blocks for sectioning and stained with hematoxylin and eosin (H&E). The immunohistochemical markers were CD117, CD34, and DOG-1, while gene testing examined the gene mutation in the KIT exons 9, 11, 13, and 17, and PDGFRA exons 12, 14, and 18 with the patient's consent. However, two cases (the series numbers are 11 and 28 in Supplementary File 1) were diagnosed as EGIST by skilled pathologists without immunohistochemical markers and gene testing. Combinating with no history of tumor resection, an isolated primary lesion, perioperative data, and pathological evidence, we diagnosed two cases as EGIST [14].

### 2.1. Statistical Analysis

Propensity score matching (PSM) was used to balance the difference between EGIST and GIST data in baseline characteristics (but surgical time and surgical bleeding were not included) [18]. We further generated a Cox proportional hazards model to associate tumor location with prognosis. In the Cox model, we performed univariate analysis and screened for factors with *P* < 0.2 or when considered clinically significant. These factors were included in the multivariate Cox model for searching and validating the association between EGIST and survival outcome. Additionally, Kaplan–Meier survival curves were used to evaluate the difference between EGIST and GIST to investigate the association of EGIST with a worse prognosis. To illustrate the difference between EGIST and GIST regardless of high risk factors, we performed subgroup analysis using adjuvant therapy, tumor size, tumor necrosis, and tumor mitosis as subgroups.

To improve rigorousness, reliability, and stability of the results and to investigate potential effect modifiers, we added two sets of parallel analysis. The same set of analyses was performed when the outcome variables were OS or DFS. Similarly, we analyzed the data before and after PSM. Furthermore, we conducted sensitivity analysis using a confounding function approach, which showed the degree of the entire effect of all unmeasured and unrecognized confounders [19, 20]. For a limited sample case, we only performed the sensitivity analysis in the overall data and not in the subgroup data. In the confounding function analysis, we only discussed the 5-year OS and DFS, which agrees with the observation endpoints of most tumor studies. All analyses were performed using the statistical software R (version 3.61). All tests were 2-tailed, and statistical significance was set at *P* < 0.05.

## 3. Results

There were 62 EGIST cases from four centers and 570 GIST cases from the Guangdong Province People's Hospital; 55 EGIST and 221 GIST patients were eventually included in the analysis. A summary of the baseline characteristics of the EGIST and GIST groups before and after PSM is presented in Table 1.

Regardless of whether before or after PSM, EGIST was similar to GIST in terms of sex, ECOG, and age. Before PSM, EGIST tended to have worse factors compared to GIST (tumor size >10 cm: 60.0% vs. 17.6%; tumor necrosis “Yes”: 60.0% vs. 24.4%; tumor mitosis >5/50 HPF: 45.5% vs. 38.0%, respectively), suggesting that EGIST may be a worse malignant tumor. The median surgical time was 152 min in EGIST and 136 min in GIST. The median surgical bleeding was 100 mL in EGIST and 50 mL in GIST. There was an obvious difference between the two, suggesting that EGIST resection is more difficult than GIST, owing to some special location.

**Figure 1:**
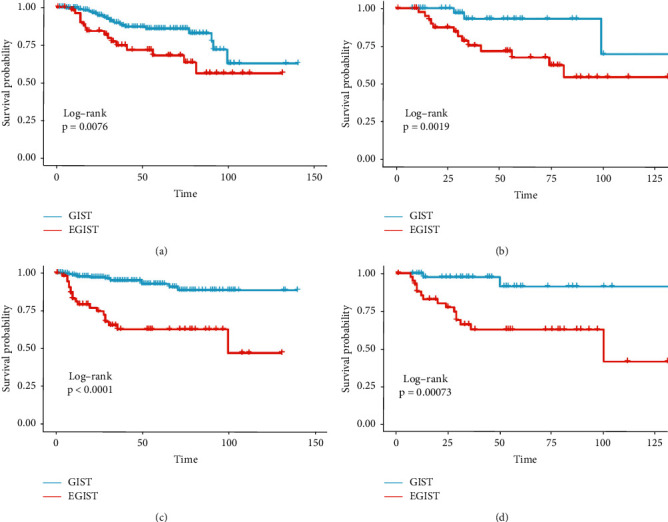
Kaplan–Meier curves comparing EGIST with GIST for overall survival. Outcomes were OS (a, c) and DFS (b, d). The outcome before (a, b) and after (c, d) propensity score matching.

We compared the EGIST and GIST data to explore the prognostic factors between them. In the univariate Cox regression model with OS as an outcome variable, tumor size was associated with a significantly worse prognosis (before PSM: HR, 2.28, 95% CI, 1.23–4.23; after PSM: HR, 2.90, 95% CI, 1.01–8.37). However, before and after PSM, there were no differences in the multivariate Cox regression model with OS as an outcome variable (Tables 2 and 3). Surprisingly, tumor size was not related to DFS in the univariate or multivariate Cox regression models after PSM (Table 3). Tumor necrosis was a key risk factor for not including EGIST data in previous studies [21], which was inconsistent with the current finding that tumor necrosis was not an independent risk factor in the multivariate Cox regression model (Tables 2 and 3). In any case, tumor location was negatively correlated with OS (before PSM in multivariate Cox regression model: HR, 2.43, 95% CI, 1.13–5.22; after PSM in multivariate Cox regression model: HR, 4.32, 95% CI, 1.22–15.26) or DFS (before PSM in multivariate Cox regression model: HR, 4.79, 95% CI, 2.20–10.43; after PSM in multivariate Cox regression model: HR, 9.79, 95% CI, 2.22–43.31), which was also intuitively shown in the Kaplan–Meier survival curves (Figure 1). Other factors associated with GIST with OS or DFS in previous analysis were not statistically significant after PSM; thus, we will inevitably think that tumor location (whether EGIST or GIST) has too much influence on OS or DFS to cover up other factors.

**Figure 2:**
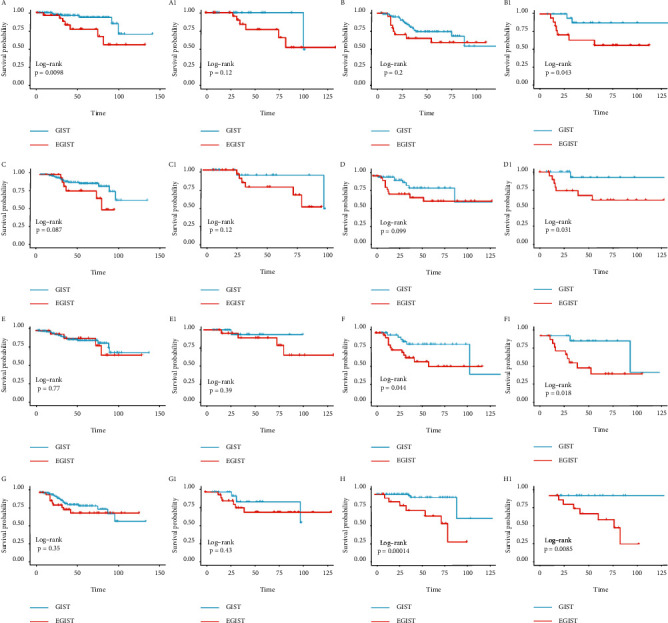
Kaplan–Meier curves comparing EGIST with GIST for subgroup. The outcome was OS. A number is not added (as, A B…) to the alphabet before propensity score matching, but a number is added (likely, A1, B1…) to the alphabet after propensity score matching. The subgroup is as follows: Tumor mitosis >5/50 HPF B B1) and Tumor mitosis ≤5/50 HPF A A1); Tumor necrosis Yes D D1) and Tumor necrosis No C C1); Tumor size >10 cm F F1) and Tumor size ≤10 cm E E1); Adjuvant therapy Accepted H H1) and Adjuvant therapy Not Accepted G G1).

**Figure 3:**
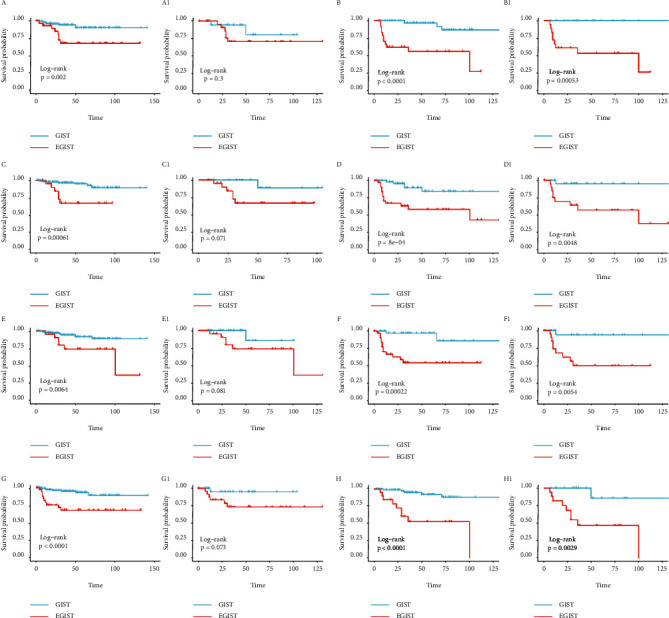
Kaplan–Meier curves comparing EGIST with GIST for subgroup. The outcome was DFS. A number is not added (as, A B…) to the alphabet before propensity score matching, but a number is added (likely, A1, B1…) to the alphabet after. The subgroup is as follows: Tumor mitosis >5/50 HPF B B1) and Tumor mitosis ≤5/50 HPF A A1); Tumor necrosis Yes D D1) and Tumor necrosis No C C1); Tumor size >10 cm F F1) and Tumor size ≤10 cm E E1); Adjuvant therapy Accepted H H1) and Adjuvant therapy Not Accepted G G1).

Subgroup analysis was conducted to discuss in depth the association between tumor location and factors such as tumor size, tumor mitosis, tumor necrosis, and adjuvant therapy (Figures 2 and 3). When tumor size in the subgroup was the “< 10 cm” group, when tumor mitosis as subgroup was the “< 5/50 HPF” group, and when tumor necrosis as subgroup was the “yes” group, there were no differences in OS or DFS after PSM. However, in the opposite subgroup, EGIST had a worse prognosis whether the outcome was OS or DFS and regardless whether after or before PSM (Figures 2 and 3).

When adjuvant therapy as a subgroup was in the “no accepted” group, EGIST has similar prognosis to GIST; it might be unexpected, but EGIST showed an obviously negative association with prognosis when another subgroup was in the “accepted” group. In short, it should be affirmed that EGIST was negatively associated with prognosis compared to GIST.

To adjust for the potential effect of some unmeasured confounders, details of the results of confounding function adjustments are shown in Supplementary File 2. Although the curves in the supplementary figure intersect to a certain extent, the overall curve still tends to show all combinations of c(0) of c(1) up to a value of 2.0, none of which would bring the corrected RR to 1 for OS and DFS. Based on the supplementary figure, we can conservatively affirm that EGIST has a worse prognosis than GIST (Supplementary File 2).

## 4. Discussion

Our study demonstrates that EGIST is associated with a worse prognosis compared to GIST patients. The strength of this relationship was obvious in EGIST patients with high risk factors, including accepted adjuvant therapy, larger tumor size, tumor necrosis, and tumor mitosis, while there was a similar prognosis without those high risk factors.

Currently, some studies [2–6, 14] have shown that EGIST is likely to be a secondary tumor based on data containing only EGIST. A clinicopathologic analysis of 95 cases of omental GIST has demonstrated that many omental GIST was gastric or small intestinal GIST masquerading as omental tumors, mainly because KIT-positive Cajal cells were not found in normal omental tissue [3]. However, Zheng et al. analyzed 25 cases of EGIST from three centers in China and were in favor of EGIST as a special subtype of GIST based on molecular genetics [5]. Moreover, Yi et al. suggested that EGIST originates from precursor cells or pluripotent stem cells located outside the gastrointestinal tract [4, 22]. When a controversy identified that the origin of EGIST was continuing, exploring the prognosis features of EGIST based on EGIST data only and guiding the EGIST treatment based on the GIST pattern may be inappropriate.

In addition, several studies aiming to illustrate a worse prognosis for EGIST and its clinicopathological characteristics failed to systematically combine clinical information with the association and difference between EGIST and GIST [2–6, 14], which may in part be attributed to low incidence and focus on the controversy of whether EGIST is a primary or secondary tumor. A study [2] including 112 “GISTs” located in the retroperitoneum showed that these tumors are a heterogenous group of “GISTs,” in addition to usually having poor outcomes, and mitotic rate >50/5 mm^2^ was significant for shorter survival. However, this study was unable to demonstrate the survival difference in EGIST without high risk factors due to the lack of comparison with GIST. Similarly, another research [6] showed that the risk for adverse outcomes increased with an increasing number of negative histologic factors. More importantly, most of these studies [2–6, 14] focused on exploring risk factors in EGIST but not in both EGIST and GIST, which, to a certain extent, could not reach a consensus on the poor prognosis of EGIST compared with GIST and did not reflect the unique clinical characteristics of EGIST. Confusions exist around the survival difference between EGIST and GIST. Meanwhile, lack of awareness of the prognostic differences of EGIST for GIST under different risk factors can easily lead to over- or undertreatment [23].

The problems could be solved by combining EGIST with GIST in exploring the clinicopathological characteristics of EGIST. In this study, the propensity score-adjusted baseline characteristic difference between the EGIST and GIST cohorts was determined to perform a comparative analysis confirming that EGIST had a worse prognosis than GIST. The EGIST prognosis compared to GIST was influenced by high risk factors, including tumor size, adjuvant therapy, tumor necrosis, and tumor mitosis; therefore, we systematically sorted these factors by subgroup analysis of the EGIST versus GIST comparative study. In the tumor size (<5 cm) subgroup analysis, a previous study [6] showed that tumor size (<5 cm) has an excellent prognosis, as was seen in GIST. Although other studies also showed that tumor size was not associated with prognosis in the EGIST only cohort [3–5], there was a lack of comparison with GIST. Furthermore, tumor necrosis [6] and tumor mitosis (>5/50 HPF) [2, 5] were viewed as negatively associated with prognosis in the EGIST only cohort, suggesting that these high risk factors may be a poor turn for the malignant manifestation of EGIST. Thus, EGIST with tumor necrosis or mitosis was associated with a worse prognosis than GIST. This result in the subgroup analysis was surprisingly consistent. In the adjuvant therapy subgroup, however, the result of whether EGIST accepted adjuvant therapy was seemingly conflicting in this study. Our previous work showed that adjuvant therapy might be unable to improve patient prognosis in EGIST regardless of DFS or OS [14]; in contrast, adjuvant therapy obviously improved the GIST of DFS and OS [8, 9, 11]. This may be because EGIST shortened the coincident indication, clinicians tended to apply GIST indication to the patient, and unaccepted adjuvant therapy patients tend to lack high risk factors. Therefore, it was consistent with our conclusion that EGIST without high risk factors had the same prognosis as GIST and may be a different tumor subtype.

Our study demonstrated that EGIST without high risk factors had a similar prognosis to GIST, but high risk factors had a worse prognosis than GIST for the first time. This may be because EGIST directly or indirectly lost its connection to the gastrointestinal tract and was in an unconventional location; thus, a delay in the presentation of clinical symptoms results in most EGIST being diagnosed at an advanced stage. EGIST is resected through open or complicated surgery, which may cause accidental injury to other organs and blood vessels [24, 25]. A total of 1,056 patients with GIST who underwent surgery with curative intention showed that DFS was inconsistent between the different locations the GIST, and we conservatively regarded “Others (*n* = 50)” as “EGIST (*n* = 50)” and that EGIST was the worse outcome [13]. Similarly, Joensuu et al. [12] found that 61 of the EGIST cases had the shortest DFS relative to other sites. If these studies further analyzed the EGIST prognosis by excluding the confounding bias using balance baseline difference or subgroup analysis, it may be similar to our result. Regarding the poor prognosis under high risk factors, including accepted adjuvant therapy, larger tumor size, tumor necrosis, and tumor mitosis, it was likely that EGIST is a unique biological behavior of GIST and a local metastasis. However, a large Chinese cohort [26] found that the DFS of EGIST was worse than that of the stomach, but better than that of the rectum. This suggested that EGIST was likely to have a worse outcome and had the same prognosis as in some subgroups. Without high risk factors, EGIST prognosis was not statistically significant. A study [7] also illustrated that “Other” group had parallel DFS in the presented data. However, this study failed to perform a subgroup analysis; thus, its results did not perfectly match our outcome. EGIST should theoretically have a worse prognosis if it is a secondary tumor from GIST, and EGIST should theoretically have a worse prognosis [10]. Consequently, EGIST may be a particular GIST subtype rather than metastasis from GIST or recurrence after GIST resection.

Our study has several limitations. First, a retrospective database led to many potential biases, such as information bias and unmeasured confounding factors. Although a random controlled trial will powerfully prove the conclusion, it may not be realistic because EGIST, whose sample size was very small, had a very low incidence, had a worse prognosis, and lacked a preliminary basis. We used a propensity matching score to reduce the difference between EGIST and GIST and used a confounding function to exclude unmeasured confounding factor interference [19, 20]. Therefore, our research played an important role in showing the clinicopathologic characteristics of EGIST in comparison with GIST. Second, we were unable to identify whether EGIST was connected to the gastrointestinal tract in pathological specimens because it was difficult to reevaluate tumor margins from searching for residual gut wall tissue in the tumor through the specimen [5]. However, we confirmed that EGIST was an independent lesion without connection with other organs based on radiological imaging, history of surgery, pathological reporter, and so on [14]. Third, the molecular genetics aspect in EGIST was not analyzed and shown because its partial definition as EGIST in this study depended on attention to precise tumor location, comprehensive immunohistochemistry, and molecular gene analysis. Lastly, whether EGIST was a primary or secondary tumor needs further exploration. Nevertheless, the clinicopathologic differences between GIST and EGIST were mainly researched in this study, and combining EGIST with GIST has deepened our knowledge of EGIST, which helps in seeking an adaptable treatment.

## 5. Conclusion

While previous studies suggested a worse prognosis in EGIST and the clinicopathological characteristics of EGIST, our findings suggest that there is a similar prognosis in patients without high risk factors. Ideally, a prospective randomized controlled trial would be applied to illustrate the characteristics of EGIST compared with GIST. However, it was challenging to determine the number of cases for the trial, and observation research is still the optimal evidence to guide treatment. Our study confirmed that EGIST was associated with a poor prognosis compared to GIST patients. Recognizing that the strength of this relationship was obvious in EGIST patients with some high risk factors, including accepted adjuvant therapy, larger tumor size, tumor necrosis, and tumor mitosis, there was a similar prognosis without those high risk factors. This prompted us to evaluate patients comprehensively, and for those without risk factors, routine treatment should be followed.

## Figures and Tables

**Table 1:** Baseline characteristics before and after propensity score matching.

	No. (%)					
	Before matching			After matching		
Characteristic	GIST (*n* = 221)	EGIST (*n* = 55)	Standardized difference^*∗*^	GIST (*n* = 43)	EGIST (*n* = 43)	Standardized difference^*∗*^

Sex (%)						
Female	106(48.0)	24 (43.6)	0.087	18 (41.9)	19 (44.2)	0.047
Male	115 (52.0)	31 (56.4)		25 (58.1)	24 (55.8)	
Age (%)						
≤60 years	112 (50.7)	31 (56.4)	0.114	30 (69.8)	26 (60.5)	0.196
>60 years	109 (49.3)	24 (43.6)		13 (30.2)	17 (39.5)	
ECOG (%)						
0	67 (30.3)	11 (20.0)	0.326	9 (20.9)	11 (25.6)	0.231
1	118 (53.4)	38 (69.1)		32 (74.4)	28 (65.1)	
>1	36 (16.3)	6 (10.9)		2 (4.7)	4 (9.3)	
Tumor size (%)						
≤10 cm	182 (82.4)	22 (40.0)	0.965	24 (55.8)	22 (51.2)	0.093
>10 cm	39 (17.6)	33 (60.0)		19 (44.2)	21 (48.8)	
Tumor necrosis (%)						
Yes	54 (24.4)	33 (60.0)	0.772	22 (51.2)	21 (48.8)	0.047
No	167 (75.6)	22 (40.0)		21 (48.8)	22 (51.2)	
Tumor mitosis (%)						
≤5/50 HPF	137 (62.0)	30 (54.5)	0.151	22 (51.2)	24 (58.1)	0.140
>5/50 HPF	84 (38.0)	25 (45.5)		21 (48.8)	18 (41.9)	
Histopathological classification (%)						
Spindle	187 (84.6)	42 (76.4)	0.209	35 (81.4)	33 (76.7)	0.115
Others	34 (15.4)	13 (23.6)		8 (18.6)	10 (23.3)	
Adjuvant therapy (%)						
Accepted	77 (34.8)	20 (36.4)	0.032	19 (44.2)	17 (39.5)	0.094
Not accepted	144 (65.2)	35 (63.6)		24 (55.8)	26 (60.5)	

**Table 2:** Cox regression model to evaluate the association of EGIST with OS or DFS before propensity score matching.

Factors	HR (95% CI)			
	OS		DFS	
	Univariate analysis	Multivariate analysis	Univariate analysis	Multivariate analysis

Sex female (vs. male)	1.14 (0.62–2.11)		0.74 (0.36–1.52)	
Age >60 years (vs. ≤60 years)	1.93 (1.01–3.69)^*∗*^	1.72 (0.85–3.48)	0.98 (0.49–1.97)	
ECOG 1 (vs. ECOG 0)	1.04 (0.50–2.17)		1.02 (0.44–2.34)	
ECOG >1 (vs. ECOG 0)	0.89 (0.32–2.45)		1.17 (0.41–3.39)	
Tumor size >10 cm (vs. ≤10 cm)	2.28 (1.23–4.23)^*∗*^	1.18 (0.55–2.54)	2.69 (1.35–5.40)^*∗*^	1.36(0.63–2.94)
Tumor necrosis yes (vs. no)	1.54 (0.83–2.88)	0.74 (0.36–1.52)	2.96 (1.47–5.95)^*∗*^	
Tumor mitosis >5/50 HPF (vs. ≤5/50 HPF)	3.03 (1.59–5.79)^*∗*^	3.50 (1.68–7.28)^*∗*^	1.33 (0.66–2.66)	
Histopathological classification spindle (vs. others)	0.50 (0.26–0.97)	0.62 (0.32–1.22)	0.58 (0.27–1.26)	0.60 (0.28–1.31)
Adjuvant therapy accepted (vs. not accepted)	0.54 (0.26–1.09)	0.42 (0.20–0.89)^*∗*^	1.33 (0.66–2.67)	1.66 (0.77–3.59)
Location EGIST (vs. GIST)	2.30 (1.23–4.33)^*∗*^	2.43 (1.13–5.22)^*∗*^	6.31(3.11–12.8)^*∗*^	4.79 (2.20–10.43)^*∗*^

OS: overall survival; DFS: disease-free survival; HR: hazard ratio; CI: confidence interval, ECOG: Eastern Cooperative Oncology Group;^*∗*^*P* < 0.05.

**Table 3:** Cox regression model to evaluate the association of EGIST with OS or DFS after propensity score matching.

Factors	HR (95% CI)			
	OS		DFS	
	Univariate analysis	Multivariate analysis	Univariate analysis	Multivariate analysis

Sex female (vs. male)	1.39 (0.51–3.77)		0.85 (0.31–2.32)	
Age >60 years (vs. ≤60 years)	3.13 (1.14–8.64)^*∗*^	1.92 (0.59–6.25)	1.62 (0.63–4.21)	
ECOG 1 (vs. ECOG 0)	1.21 (0.34–4.40)		0.45 (0.16–1.28)	
ECOG >1 (vs. ECOG 0)	2.49 (0.41–15.22)		1.12 (0.22–5.70)	
Tumor size >10 cm (vs. ≤10 cm)	2.90 (1.01–8.37)^*∗*^	2.55 (0.75–8.66)	1.93 (0.73–5.08)	2.24 (0.84–5.98)
Tumor necrosis yes (vs. no)	0.95 (0.36–2.55)		1.46 (0.55–3.86)	
Tumor mitosis >5/50 HPF (vs. ≤5/50 HPF)	1.47 (0.55–3.95)		1.34 (0.51–3.47)	
Histopathological classification spindle (vs. others)	0.45 (0.16–1.24)	0.43 (0.15–1.23)	0.48 (0.18–1.29)	0.51 (0.18–1.44)
Adjuvant therapy accepted (vs. not accepted)	1.08 (0.40–2.91)		2.10 (0.80–5.53)	2.22 (0.82–6.03)
Location EGIST (vs. GIST)	4.01 (1.14–14.11)^*∗*^	4.32 (1.22–15.26)^*∗*^	8.36 (1.91–36.57)^*∗*^	9.79 (2.22–43.31)^*∗*^

OS: overall survival; DFS: disease-free survival; HR: hazard ratio; CI: confidence interval, ECOG: Eastern Cooperative Oncology Group; *P* < 0.05.

## Data Availability

The data that support the findings of this study are available from the corresponding author upon reasonable request, and Supplementary File 1 is a part of original data.

## Supplementary Materials

Supplementary Materials Supplementary File 1: the original data of the current study. Supplementary File 2: sensitivity analysis for the relative risk of OS and DFS was performed using inverse probability of treatment weighting.
