# Supplementary material for: Clinical Features of Extragastrointestinal Stromal Tumor Compared with Gastrointestinal Stromal Tumor: A Retrospective, Multicenter, Real-World Study
Source: J Oncol. 2021 Dec 13;2021:1460131. doi: 10.1155/2021/1460131 (PMC8687840; doi:10.1155/2021/1460131)
Supplement: Supplementary Materials — Supplementary File 1: the original data of the current study. Supplementary File 2: sensitivity analysis for the relative risk of OS and DFS was performed using inverse probability of treatment weighting. [file 1460131.f1.zip › 1460131.f1/Supplement file 1.pdf]

Institution	Serial number	Tumor rupture
Guangdong Provincial People’s Hospital	1	No
Guangdong Provincial People’s Hospital	2	No
Guangdong Provincial People’s Hospital	3	No
Guangdong Provincial People’s Hospital	4	No
Guangdong Provincial People’s Hospital	5	No
Guangdong Provincial People’s Hospital	6	No
Guangdong Provincial People’s Hospital	7	No
Guangdong Provincial People’s Hospital	8	No
Guangdong Provincial People’s Hospital	9	No
Guangdong Provincial People’s Hospital	10	No
Guangdong Provincial People’s Hospital	11	No
Guangdong Provincial People’s Hospital	12	No
Guangdong Provincial People’s Hospital	13	No
Guangdong Provincial People’s Hospital	14	No
Guangdong Provincial People’s Hospital	15	Yes
Guangdong Province Traditional Medical Hospital	16	No
Guangdong Province Traditional Medical Hospital	17	No
Guangdong Province Traditional Medical Hospital	18	No
Guangdong Province Traditional Medical Hospital	19	No
Guangdong Province Traditional Medical Hospital	20	No
Guangdong Province Traditional Medical Hospital	21	No
Guangdong Province Traditional Medical Hospital	22	No
Guangdong Province Traditional Medical Hospital	23	Yes
Liaoning Cancer Hospital & Institute	24	No
Liaoning Cancer Hospital & Institute	25	No
Liaoning Cancer Hospital & Institute	26	No
Liaoning Cancer Hospital & Institute	27	No
Liaoning Cancer Hospital & Institute	28	No
Liaoning Cancer Hospital & Institute	29	No
Liaoning Cancer Hospital & Institute	30	No
Liaoning Cancer Hospital & Institute	31	No
Liaoning Cancer Hospital & Institute	32	No
Liaoning Cancer Hospital & Institute	33	No
Liaoning Cancer Hospital & Institute	34	No
Liaoning Cancer Hospital & Institute	35	No
Liaoning Cancer Hospital & Institute	36	No
Liaoning Cancer Hospital & Institute	37	No
Liaoning Cancer Hospital & Institute	38	No
Liaoning Cancer Hospital & Institute	39	No
Liaoning Cancer Hospital & Institute	40	No
Liaoning Cancer Hospital & Institute	41	No
Liaoning Cancer Hospital & Institute	42	No
Liaoning Cancer Hospital & Institute	43	No
Liaoning Cancer Hospital & Institute	44	No
Liaoning Cancer Hospital & Institute	45	No
Liaoning Cancer Hospital & Institute	46	No
Liaoning Cancer Hospital & Institute	47	No
Liaoning Cancer Hospital & Institute	48	No
Fujian Medical University Union Hospital	49	No
Fujian Medical University Union Hospital	50	No
Fujian Medical University Union Hospital	51	No
Fujian Medical University Union Hospital	52	No
Fujian Medical University Union Hospital	53	No
Fujian Medical University Union Hospital	54	No
Fujian Medical University Union Hospital	55	No

Surgical margin	Retroperitoneal	tumor size (cm)	Histopathologica	Tumor necrosis
-	Yes	23	Spindle	Yes
-	No	5	Spindle	No
-	No	9	Mixed	Yes
-	No	17	Spindle	No
-	No	10	Spindle	Yes
-	No	18	Mixed	No
-	No	19	Spindle	Yes
-	No	22	Mixed	Yes
-	No	14	Spindle	Yes
-	Yes	7	Mixed	Yes
-	Yes	14	Spindle	Yes
-	No	0.4	Spindle	No
-	No	6	Spindle	No
-	No	7	Mixed	No
-	Yes	22	Mixed	Yes
-	No	12.5	Spindle	No
-	No	3	Spindle	No
-	No	0.4	Spindle	No
-	Yes	13	Mixed	Yes
-	Yes	16	Mixed	Yes
-	No	16	Spindle	Yes
-	No	22	Spindle	Yes
+	Yes	20	Mixed	No
-	No	20	Spindle	No
-	No	6	Mixed	Yes
-	No	9.5	Spindle	No
-	No	4.5	Spindle	No
-	Yes	6.5	Spindle	No
-	No	13	Spindle	Yes
-	No	7	Spindle	No
-	No	16	Spindle	Yes
-	Yes	15	Spindle	Yes
-	Yes	14	Spindle	Yes
-	No	29	Mixed	No
-	Yes	17	Spindle	Yes
-	Yes	7	Spindle	Yes
-	No	18	Spindle	Yes
-	Yes	29	Spindle	Yes
-	Yes	9	Spindle	No
-	Yes	11	Spindle	No
-	Yes	7	Spindle	Yes
-	No	6	Spindle	No
-	Yes	5	Epithelioid	No
-	No	12	Spindle	Yes
-	No	6	Spindle	No
-	Yes	13	Spindle	Yes
-	No	20	Spindle	Yes
-	No	15	Spindle	Yes
+	No	6.5	Spindle	Yes
-	No	9	Spindle	Yes
-	Yes	25	Mixed	Yes
-	No	16	Spindle	Yes
-	Yes	14	Spindle	Yes
-	No	23	Spindle	Yes
+	No	2	Spindle	No

CD117	CD34	DOG1	Gene mutation	KIT.9	KIT.11	KIT.13	KIT.17
positive	positive	unknown	none detected				
positive	positive	positive	Mutant	unknown	Mutant	unknown	unknown
positive	negative	unknown	none detected				
positive	positive	unknown	none detected				
positive	positive	positive	Mutant	Wild	Wild	Wild	Wild
positive	negative	unknown	none detected				
positive	positive	unknown	none detected				
positive	negative	unknown	none detected				
positive	positive	positive	Mutant	unknown	Mutant	unknown	unknown
positive	positive	negative	Mutant	Mutant	unknown	unknown	unknown
negative	negative	unknown	none detected				
positive	positive	positive	none detected				
positive	positive	unknown	none detected				
positive	negative	negative	Mutant	Wild	Wild	Wild	Wild
positive	positive	unknown	Mutant	unknown	Mutant	unknown	unknown
negative	positive	unknown	none detected				
negative	positive	unknown	none detected				
positive	positive	unknown	none detected				
positive	positive	unknown	none detected				
positive	negative	unknown	none detected				
positive	negative	unknown	none detected				
positive	negative	unknown	none detected				
positive	negative	unknown	none detected				
positive	positive	positive	Mutant	Wild	Wild	Wild	Wild
positive	positive	positive	Mutant	unknown	unknown	unknown	unknown
positive	positive	positive	Mutant	unknown	unknown	unknown	unknown
positive	positive	positive	Mutant	unknown	Mutant	unknown	unknown
negative	negative	negative	none detected				
positive	positive	positive	none detected				
positive	positive	positive	Mutant	unknown	Mutant	unknown	unknown
positive	positive	positive	Mutant	Mutant	unknown	unknown	unknown
negative	positive	positive	none detected				
positive	negative	unknown	Mutant	unknown	Mutant	unknown	unknown
positive	positive	positive	none detected				
negative	positive	negative	Mutant	unknown	Mutant	unknown	unknown
positive	positive	positive	none detected				
positive	positive	negative	Mutant	unknown	Mutant	unknown	unknown
negative	positive	negative	none detected				
positive	positive	negative	none detected				
positive	positive	positive	Mutant	unknown	Mutant	unknown	unknown
positive	positive	positive	none detected				
negative	negative	positive	Mutant	unknown	unknown	unknown	unknown
positive	positive	negative	Mutant	unknown	Mutant	unknown	unknown
negative	positive	negative	Mutant	unknown	unknown	unknown	unknown
negative	positive	negative	none detected				
negative	negative	negative	Mutant	unknown	unknown	unknown	unknown
positive	negative	positive	Mutant	unknown	Mutant	unknown	unknown
negative	positive	negative	Mutant	unknown	Mutant	unknown	unknown
positive	positive	negative	Mutant	Wild	Wild	Wild	Wild
positive	negative	positive	none detected				
positive	negative	positive	none detected				
positive	positive	positive	Mutant	unknown	Mutant	unknown	unknown
positive	positive	negative	none detected				
positive	positive	positive	none detected				
positive	positive	positive	none detected				

PDGFRA.1	PDGFRA.18
unknown	unknown
Wild	Wild
unknown	unknown
unknown	unknown
Wild	Wild
unknown	unknown
Wild	Wild
unknown	Mutant
unknown	Mutant
unknown	unknown
unknown	unknown
unknown	unknown
unknown	unknown
unknown	unknown
unknown	unknown
unknown	unknown
Mutant	unknown
unknown	unknown
unknown	unknown
unknown	unknown
unknown	unknown
unknown	unknown
Wild	Wild
unknown	unknown
