# Supplementary material for: Clinical Features of Extragastrointestinal Stromal Tumor Compared with Gastrointestinal Stromal Tumor: A Retrospective, Multicenter, Real-World Study
Source: J Oncol. 2021 Dec 13;2021:1460131. doi: 10.1155/2021/1460131 (PMC8687840; doi:10.1155/2021/1460131)
Supplement: Supplementary Materials — Supplementary File 1: the original data of the current study. Supplementary File 2: sensitivity analysis for the relative risk of OS and DFS was performed using inverse probability of treatment weighting. [file 1460131.f1.zip › 1460131.f1/supplement file 2.pdf]

Introduction: Confounding function

We performed sensitivity analysis using a confounding function approach, which described the degree of the entire effect of all unmeasured and unrecognized confounders. The relative risks (RRs) of OS and DFS were calculated using a log binomial model with inverse probability of treatment weighting (IPTW) using the propensity score. The marginal RRs obtained from IPTW were corrected for various values of confounding functions, c(0) and c(1). These parameters are counterfactual; c(0) represents a hypothetical RR of the outcome (OS or DFS) comparing those who actually did and did not was EGIST, but assuming none was EGIST, while c(1) represents a hypothetical RR of the outcome comparing those who actually did and did was EGIST, but assuming all was EGIST. The values of c(0) and c(1)were then varied to estimate the effect of unmeasured confounding required to provide an adjusted RR. For example, c(0) = c(1) assumes that there are no unmeasured effect modifiers, c(0) > 1 and c(1) > 1 implies that the exposed participants would have had a greater risk of the potential outcome than unexposed participants if they had the same exposure status.

Result: Analysis of the potential effect of unmeasured confounders

The RRs obtained using IPTW were (a) 2.16 for OS and (b) 4.47 for DFS. In sensitivity analyses (figures are as follow), these RRs were corrected for hypothetical confounding functions c(0) and c(1), which showed that RRs decreased as the values of the confounding functions increased. In the absence of effect modifiers and unmeasured confounders, the value of c(1) = c(0) equals 1. For example, for OS, assuming c(0) = c(1), the unmeasured confounding-adjusted RR would be equal to 1 if the confounding function was at least 2.16 (that is, patients with EGIST would have to be at least 2.16 times more likely to have OS than the controls, if they had the same exposure status, to remove the entire observed association between EGIST and OS). The figure as follows shows all combinations of c(0) = c(1) up to a value of 2.0, none of which would bring the corrected RR to 1 for OS.

a. Overall Survival (OS)

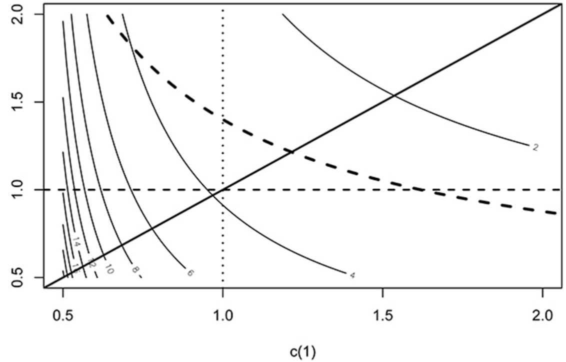

b. Disease-Free Survival (DFS)

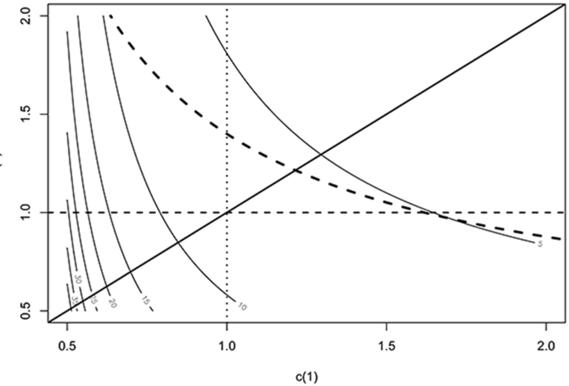

Sensitivity analysis for the RR of OS and DFS was performed using IPTW. The marginal RRs obtained from IPTW were corrected for various values of confounding functions, c(0) and c(1). These parameters are counterfactual, where c(1) represents a hypothetical RR of the outcome (OS or DFS) compared to those who actually were or were not EGIST, but assuming all was EGIST. The values of c(0) and c(1) were then varied to estimate the effect of unmeasured confounding required to provide an adjusted RR. For example, c(0) = c(1) denotes the assumption that there were no unmeasured effect modifiers, and c(0) > 1 and c(1) > 1 implies that exposed participants would have had a greater risk of the potential outcome than unexposed participants, who had the same exposure status. The solid straight line indicates c(0) = c(1). When c(0) = (c1) = 1, the unadjusted relative risk for unmeasured confounding is (a) 2.16 for OS and (b) 4.47 for DFS. The dashed curve indicates the value of c(0) and (1) required to give a relative risk of (a) 2.16 for OS and (b) 4.47 for DFS.
